# Hesperetin-5,7,3’-*O*-triacetate suppresses airway hyperresponsiveness in ovalbumin-sensitized and challenged mice without reversing xylazine/ketamine-induced anesthesia in normal mice

**DOI:** 10.1186/s40360-017-0146-5

**Published:** 2017-05-30

**Authors:** You-Lan Yang, Chi-Li Chen, Chi-Ming Chen, Wun-Chang Ko

**Affiliations:** 1 School of Respiratory Therapy, College of Medicine, Taipei Medical University, Taipei, Taiwan; 2 Department of Medicinal Chemistry, College of Pharmacy, Taipei Medical University, Taipei, Taiwan; 3 Department of Pharmacology, College of Medicine, Taipei Medical University, Taipei, Taiwan

**Keywords:** Airway hyperresponsiveness, Airway resistance, Hesperetin-5,7,3’-*O*-triacetate, Lung dynamic compliance, Roflumilast, Xylazine/ketamine-induced anesthesia

## Abstract

**Background:**

We recently reported that hesperetin-5,7,3’-*O*-triacetate (HTA) dually inhibited phosphodiesterase (PDE)3/4 with a therapeutic ratio of 20.8. The application and development of PDE4 inhibitors for treating asthma or COPD are limited by their side effects, such as nausea, vomiting and gastric hypersecretion. PDE4 inhibitors were reported to reverse xylazine/ketamine-induced anesthesia in rats and triggered vomiting in ferrets. Thus the reversing effect of HTA on xylazine/ketamine-induced anesthesia in mice was studied to assess emetic effect of HTA. The aim of this study was to prove the therapeutic effect of HTA without vomiting effect at an effective dose for treating COPD.

**Methods:**

Ten female BALB/c mice in each group were sensitized by ovalbumin (OVA) on days 0 and 14. On day 21, these mice were emphasized the sensitization by Freund’s complete adjuvant. Mice were challenged by 1% OVA nebulization on days 28, 29, and 30. Airway hyperresponsiveness (AHR) was assessed on day 32 in each group, using the FlexiVent system to determine airway resistance (R_L_) and lung dynamic compliance (C_dyn_) in anesthetized ovalbumin (OVA)-sensitized and challenged mice. Each group was orally administered HTA (10 ~ 100 μmol/kg), roflumilast (1 and 5 mg/kg) or vehicles (controls) 2 h before and 6 and 24 h after OVA provocation. For comparison, sham-treated mice were challenged with saline instead of 1% OVA. The ability to reverse xylazine/ketamine-induced anesthesia by HTA or roflumilast for 3 h was determined in normal mice. We used roflumilast, a selective PDE4 inhibitor and bronchodilator for severe COPD approved by the US Food and Drug Administration, as a reference drug.

**Results:**

In the results, HTA (100 μmol/kg, p.o.) or roflumilast (5 mg/kg, p.o.) significantly suppressed all R_L_ values of MCh at 0.78 ~ 25 mg/mL and enhanced C_dyn_ values of MCh at 3.125 ~ 25 mg/mL compared to OVA-sensitized and -challenged control mice. Orally administered 1, 3 or 10 mg/kg roflumilast, but not 30 or 100 μmol/kg HTA, significantly reversed xylazine/ketamine-induced anesthesia.

**Conclusions:**

In contrast to roflumilast, HTA may ameliorate COPD but induce few side effects of nausea, vomiting and gastric hypersecretion at an effective dose for treating COPD, because HTA did not reverse xylazine/ketamine-induced anesthesia in mice.

## Background

It is known that phosphodiesterases (PDEs) comprise at least 11 distinct enzyme families that hydrolyze adenosine 3′,5′ cyclic monophosphate (cAMP) and/or guanosine 3′,5′ cyclic monophosphate (cGMP) [1]. PDE3 and PDE4 families are cGMP-inhibited and cAMP-specific, respectively. PDE4 may have high (PDE4_H_) and low (PDE4_L_) affinities for rolipram. In general, it is believed that inhibition of PDE4_H_ is associated with adverse responses, such as nausea, vomiting, and gastric hypersecretion, while inhibition of PDE4_L_ is associated with anti-inflammatory and bronchodilating effects. Therefore, the therapeutic ratio of selective PDE4 inhibitors for treating asthma and chronic obstructive pulmonary disease (COPD) is defined as the PDE4_H_/PDE4_L_ ratio [2].

Hesperetin (5,7,3’-trihydroxy-4’-methoxyflavanone) was reported to selectively inhibit PDE4 activity [3], and is used as a lead compound to synthesize hesperetin-5,7,3’-*O*-triacetate (HTA), a more-liposoluble derivative of hesperetin. HTA was reported to dually inhibit PDE3/4 with a therapeutic (PDE4_H_/PDE4_L_) ratio of 20.8 [4], which is greater than that of roflumilast [5], a selective PDE4 inhibitor. Roflumilast was approved by the European Commission [6], and the US Food and Drug Administration (FDA) [4] as an adjunct to bronchodilator therapy for severe COPD associated with chronic bronchitis in adults with a history of frequent exacerbations. However, dual PDE3/4 inhibitors are reported to have additive or synergistic anti-inflammatory and bronchodilator effects compared to PDE3 or PDE4 inhibitors alone [7]. In other words, the real therapeutic ratio of dual PDE3/4 inhibitors should be greater than that reported [4]. Therefore, we were interested in investigating the suppressive effects of HTA on ovalbumin (OVA)-induced airway hyperresponsiveness (AHR), and clarifying its potential for treating atypical asthma and COPD [8]. In this animal model, the number of neutrophils in the bronchoalveolar lavage fluid of control sensitized and challenged mice was significantly greater than that of eosinophils [8]. AHR was previously assessed by barometric plethysmography [9] using a whole-body plethysmograph in unrestrained animals. However, the determination of enhanced pause does likely not reflect lung mechanics [10, 11]. Thus AHR in the present study was assessed using the FlexiVent system to determine the airway resistance (R_L_) and lung dynamic compliance (C_dyn_) in anesthetized ventilated mice. The application and development of PDE4 inhibitors for treating asthma and COPD are limited by their side effects, such as nausea, vomiting and gastric hypersecretion [2]. PDE4 inhibitors were reported to reverse xylazine/ketamine-induced anesthesia in rats [12] and triggered vomiting in ferrets [13]. Thus the reversing effect of HTA on xylazine/ketamine-induced anesthesia in mice was used to assess emetic effect of HTA. The aim of this study was to prove the therapeutic effect of HTA without vomiting effect at effective dose for treating COPD. To compare the therapeutic and gastrointestinal (GI) side effects of HTA, roflumilast was used as a reference drug.

## Methods

### Reagents and animals

**Fig. 1:**
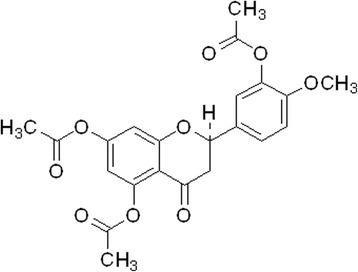
Chemical structure of hesperetin-5,7,3’-*O*-triacetate (HTA; mol. wt., 428.27)

HTA (mol. wt., 428.27, Fig. 1) was synthesized in accordance with a previously described method [14]. The purity of HTA exceeded 98% and the structure was determined by spectral methods [4]. The reference drug, roflumilast (Daxas® film-coated tablets) was a gift from Takeda Pharmaceutical (Taipei, Taiwan). Aluminum sulfate hexadecahydrate, methacholine (MCh), OVA, urethane, chloralose, ethylenediaminetetraacetic acid (EDTA), dimethyl sulfoxide (DMSO), bis-tris, 3,3′,5,5′-tetramethylbenzidine (TMB) solution, xylazine hydrochloride and (±)-ketamine hydrochloride were purchased from Sigma-Aldrich Chemical (St. Louis, Missouri, USA). Freund’s adjuvant (*Mycobacterium butyricum*) was purchased from Pierce Biotechnology (Rockford, Illinois, USA). Ethyl alcohol and polyethylene glycol (PEG) 400 were purchased from Merck (Darmstadt, Germany). HTA was dissolved in a mixture of ethyl alcohol and DMSO (1: 1), whereas roflumilast was suspended in phosphate-buffered saline (PBS). Other reagents were dissolved in distilled water. The oral dosages of HTA and roflumilast were expressed as μmol/kg and mg/kg, respectively.

Female BABL/c mice at 8 ~ 12 weeks old were purchased from the Animal Center of the Ministry of Science and Technology (Taipei, Taiwan), housed in ordinary cages at 22 ± 1 °C with a humidity of 50% ~ 60% under a constant 12/12-h light/dark cycle and provided with OVA-free food and water ad libitum [8]. Under a protocol approved (LAC-100-0152) on May 4, 2012 by the Animal Care and Use Committee of Taipei Medical University, the following experiments were performed.

### AHR in vivo

In accordance with a previously published protocol [8], ten female BALB/c mice in each group were sensitized by an intraperitoneal (i.p.) injection of 20 μg of OVA emulsified in 2.25 mg of an aluminum hydroxide gel, prepared from aluminum sulfate hexadecahydrate, in a total volume of 100 μL on days 0 and 14. On day 21, these mice were (i.p.) injected with 100 μL of a mixture of 1% OVA and Freund’s complete adjuvant (1:1). Mice were challenged via the airway using 1% OVA in saline for 30 min on days 28, 29, and 30 by ultrasonic nebulization. After the last OVA challenge [15], AHR was assessed on day 32 (48 h after 1% OVA provocation) in each group. Each group of mice was orally (p.o.) administered HTA (10 ~ 100 μmol/kg), roflumilast (1 and 5 mg/kg) or vehicles (controls) 2 h before and 6 and 24 h after OVA provocation. For comparison, sham-treated mice were challenged with saline instead of 1% OVA (non-challenged). A mixture of DMSO: ethyl alcohol: PEG 400: saline (0.5: 0.5: 1: 8, v/v) or PBS was the vehicle for the control of HTA or roflumilast, respectively. The vehicles were administered (p.o.) at a volume of 0.01 mL/g of body weight. Mice showed no abnormal behavior after oral administration of the vehicle.

In accordance with a previously described method [8], anesthetized (urethane 600 mg/kg and chloralose 120 mg/kg, i.p.), tracheostomized (stainless-steel cannula, 18 G) mice were mechanically ventilated (at 150 breaths/min, with a tidal volume of 10 mL/kg and a positive end-expiratory pressure of 3 cmH_2_O). Prior to PBS nebulization for 10 s the baseline R_L_ and C_dyn_ were determined. Then the AHR of mice was assessed by measuring changes in the R_L_ and C_dyn_ after being challenged with aerosolized MCh (0.78, 1.563, 3.125, 6.25, 12.5, and 25 mg/mL) for 10 s using the FlexiVent system (SCIREQ, Montreal, Quebec, Canada), in which these data were automatically saved for 3 min after 10 s of nebulization.

### Xylazine/Ketamine-induced anesthesia

According to previously reported methods [8, 16], after loss of the righting reflex (i.e., when a mouse remains on its back and no longer spontaneously rights itself to a prone position), the duration of anesthesia was measured until its return as the endpoint. The ability to reverse xylazine/ketamine-induced anesthesia by oral administration of HTA, roflumilast or their vehicles for 3 h was determined in female BALB/c mice.

### Statistical analysis

Differences among values given as the mean ± standard error of the mean (SEM) were calculated by a one-way analysis of variance (ANOVA), and then determined by Dunnett’s test. The difference between two values, however, was determined by Student’s *t*-test. Significance was accepted when *p* < 0.05.

## Results

### Suppression of AHR in vivo

**Fig. 2:**
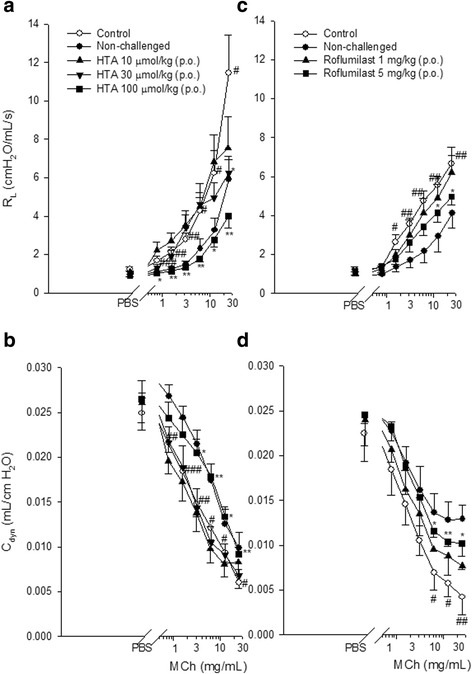
Effect of orally administered HTA (10 ~ 100 μmol/kg) and roflumilast (1 and 5 mg/kg) on the airway resistance (R_L_) (**a**, **c**) and lung dynamic compliance (C_dyn_) (**b**, **d**) in sensitized and challenged mice which received aerosolized methacholine (MCh, 6.25 ~ 25 mg/mL) 2 days after the last allergen challenge. *p < 0.05, **p < 0.01, ***p < 0.001 compared to the control (vehicle) group. ^#^ *p* < 0.05, ^##^ *p* < 0.01, ^###^ *p* < 0.001 compared to the non-challenged group. Each value represents the mean ± SEM (*n* = 5 ~ 12)

Baseline R_L_ values of control, non-challenged, and HTA-treated (10, 30, and 100 μmol/kg) groups of sensitized and challenged mice were 1.06 ± 0.08, 0.96 ± 0.07, 1.03 ± 0.06, 0.90 ± 0.10, and 0.85 ± 0.06 cmH_2_O/mL/s, which did not significantly differ from each other. After PBS nebulization, the R_L_ values of each group were 1.24 ± 0.14, 0.97 ± 0.06, 1.09 ± 0.06, 0.96 ± 0.12, and 0.90 ± 0.13 cmH_2_O/mL/s, which did not significantly differ from each other or from the respective baseline R_L_ values, suggesting that PBS nebulization did not influence baseline R_L_ values. However, MCh (0.78 ~ 25 mg/mL) concentration-dependently and significantly increased R_L_ values in sensitized and challenged control mice compared to non-challenged mice (Fig. 2a). HTA at 30 μmol/kg (p.o.) significantly suppressed the R_L_ value from 11.46 ± 1.96 to 6.25 ± 0.87 cmH_2_O/mL/s of MCh at 25 mg/mL. Furthermore, HTA 100 μmol/kg (p.o.) significantly suppressed all R_L_ values from 1.68 ± 0.22 to 1.01 ± 0.06, from 2.14 ± 0.25 to 1.13 ± 0.09, from 2.77 ± 0.37 to 1.32 ± 0.08, from 4.28 ± 0.37 to 1.78 ± 0.14, from 6.24 ± 1.19 to 2.76 ± 0.36, and from 11.46 ± 1.96 to 4.01 ± 0.62 cmH_2_O/mL/s of MCh at 0.78 ~ 25 mg/mL (Fig. 2a). In contrast, baseline C_dyn_ values of each group were 0.026 ± 0.0012, 0.030 ± 0.0017, 0.024 ± 0.0005, 0.027 ± 0.0008 and 0.027 ± 0.0022 mL/cmH_2_O, which did not significantly differ from each other (Fig. 2b). After PBS nebulization, C_dyn_ values of each group were 0.025 ± 0.0011, 0.029 ± 0.0014, 0.026 ± 0.0031, 0.026 ± 0.0008 and 0.027 ± 0.0021 mL/cmH_2_O, which did not significantly differ from each other or from the respective baseline C_dyn_ values, suggesting that PBS nebulization also did not influence baseline C_dyn_ values. However, MCh (0.78 ~ 25 mg/mL) concentration-dependently and significantly decreased C_dyn_ values in sensitized and challenged control mice compared to non-challenged mice (Fig. 2b). HTA 100 μmol/kg (p.o.) significantly enhanced C_dyn_ values from 0.015 ± 0.0015 to 0.021 ± 0.0016, from 0.012 ± 0.0013 to 0.018 ± 0.0014, from 0.009 ± 0.0011 to 0.013 ± 0.0011, and from 0.006 ± 0.0006 to 0.009 ± 0.0007 mL/cmH_2_O of MCh at 3.125 ~ 25 mg/mL when compared to sensitized and challenged control mice (Fig. 2b).

Baseline R_L_ values of control, non-challenged, roflumilast-treated (1 and 5 mg/kg) groups of sensitized and challenged mice were 1.95 ± 0.99, 0.93 ± 0.05, 1.01 ± 0.10 and 1.03 ± 0.10 cmH_2_O/mL/s, which did not significantly differ from each other. After PBS nebulization, R_L_ values of each group were 1.22 ± 0.14, 0.90 ± 0.06, 1.01 ± 0.11 and 1.15 ± 0.22 cmH_2_O/mL/s, which did not significantly differ from each other or from the respective baseline R_L_ values, suggesting that PBS nebulization did not influence baseline R_L_ values. However, MCh (1.56 ~ 25 mg/mL) concentration-dependently and significantly increased R_L_ values in sensitized and challenged control mice compared to non-challenged mice (Fig. 2c). Roflumilast at 5 mg/kg (p.o.) significantly suppressed the R_L_ values from 5.56 ± 0.41 to 4.15 ± 0.50, and from 6.65 ± 0.42 to 4.97 ± 0.42 cmH_2_O/mL/s of MCh at 12.5 and 25 mg/mL. In contrast, respective baseline C_dyn_ values of each group were 0.004 ± 0.0201, 0.025 ± 0.0009, 0.026 ± 0.0022, and 0.027 ± 0.0026 mL/cmH_2_O, which did not significantly differ from each other (Fig. 2d). After PBS nebulization, C_dyn_ values of each group were 0.023 ± 0.0031, 0.025 ± 0.0009, 0.023 ± 0.0020 and 0.026 ± 0.0022 mL/cmH_2_O, which did not significantly differ from each other or from respective baseline C_dyn_ values, suggesting that PBS nebulization also did not influence baseline C_dyn_ values. However, MCh (6.25 ~ 25 mg/mL) concentration-dependently and significantly decreased C_dyn_ values in sensitized and challenged control mice compared to non-challenged mice (Fig. 2d). Roflumilast at 5 mg/kg (p.o.) significantly enhanced C_dyn_ values from 0.007 ± 0.002 to 0.012 ± 0.001, from 0.006 ± 0.001 to 0.011 ± 0.001, and from 0.004 ± 0.002 to 0.009 ± 0.001 mL/cmH_2_O of MCh at 6.25 ~ 25 mg/mL compared to sensitized and challenged control mice (Fig. 2d).

### Xylazine/Ketamine-induced anesthesia

**Fig. 3:**
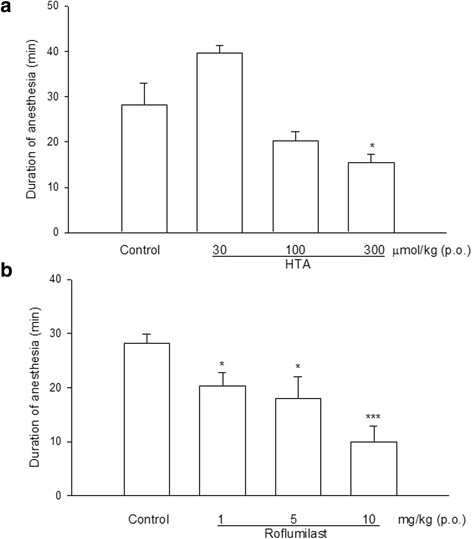
Effects of orally administered HTA (**a**) and roflumilast (**b**) on the duration of xylazine (10 mg/kg, i.p.)/ketamine (70 mg/kg, i.p.)-induced anesthesia in mice. * *p* < 0.05, *** *p* < 0.001, compared to the control. Each value represents the mean ± SEM. The number of mice in each group was 5 ~ 8

Durations of xylazine/ketamine-induced anesthesia in vehicle (control)-treated mice for the HTA- and roflumilast-treated groups were 28.2 ± 4.7 (*n* = 5) and 28.3 ± 1.7 (*n* = 8) min, respectively. Oral administration of HTA 300 μmol/kg significantly shortened the duration to 15.4 ± 1.9 (*n* = 5) min (Fig. 3a), and so did roflumilast 1, 3, and 10 mg/kg to 20.3 ± 2.48, 18.0 ± 4.07, and 10.0 ± 2.94 min, respectively (Fig. 3b).

## Discussion

HTA dually inhibits PDE3/4, whereas roflumilast selectively inhibits PDE4 activity. Thus degradation of cAMP, an important secondary messenger, is prevented by them and the intracellular cAMP content indirectly increases [15, 17–19]. Increased cAMP activates cAMP-dependent protein kinase, inhibits myosin light-chain kinase, and results in bronchodilation. Thus the R_L_ decreased and the C_dyn_ was enhanced. These results suggest that HTA would have benefits in treating COPD, although no evidence was found to support it having benefits for treating atypical asthma.

The application and development of PDE4 inhibitors in treating asthma and COPD are limited by their side effects, such as nausea, vomiting and gastric hypersecretion [2]. Rolipram, a first generation PDE4 inhibitor, has a therapeutic ratio of 0.002 [20] and has many side effects. Cilomilast and roflumilast have therapeutic ratios of 1 and 3, respectively [5, 21]. Recently, roflumilast was approved by the European Commission [6] and the US FDA [4] as an add-on to bronchodilator therapy for maintenance treatment of severe COPD associated with chronic bronchitis in adults with a history of frequent exacerbations.

Robichaud et al. reported that MK-912, an α_2_-adrenoceptor antagonist, reversed xylazine/ketamine-induced anesthesia in rats [12] and triggered vomiting in ferrets [13]. In contrast, clonidine, an α_2_-adrenoceptor agonist, prevented emesis in ferrets [13]. Thus they suggested that the reversing effect occurred through presynaptic α_2_-adrenoceptor inhibition [13]. They also found that PDE4 inhibitors reversed xylazine/ketamine-induced anesthesia in rats and ferrets [12, 13]. Thus the reversing effect of PDE4 inhibitors on xylazine/ketamine-induced anesthesia in rats or mice is convenient and could be a surrogate for assessing the emetic effects of these drugs, as rodents have no emetic reflex and we cannot observe emesis. In the present results, orally administered HTA at 300 μmol/kg (approximately 128.5 mg/kg) and roflumilast at 1 ~ 10 mg/kg significantly reversed xylazine/ketamine-induced anesthesia in mice, whereas orally administered HTA at 100 μmol/kg or roflumilast at 5 mg/kg significantly reduced the R_L_ and enhanced the C_dyn_. HTA even at 30 μmol/kg also reduced the R_L_, although did not enhance the C_dyn_.

## Conclusions

In contrast to roflumilast, HTA may ameliorate COPD but induce few side effects of nausea, vomiting and gastric hypersecretion at a dose effective for treating COPD, because HTA did not reverse xylazine/ketamine-induced anesthesia in mice.

## Abbreviations

AHR: Airway hyperresponsiveness
cAMP: Adenosine 3′,5′ cyclic monophosphate
C_dyn_: Lung dynamic compliance
cGMP: Guanosine 3′,5′ cyclic monophosphate
COPD: Chronic obstructive pulmonary disease
DMSO: Dimethyl sulfoxide
EDTA: ethylenediaminetetraacetic acid
FDA: Food and Drug Administration
GI: Gastrointestinal
HTA: Hesperetin-5,7,3’-*O*-triacetate
MCh: Methacholine
OVA: Ovalbumin
PBS: Phosphate-buffered saline
PDE: Phosphodiesterase
PDE4_H_: PDE4 high affinity for rolipram
PDE4_L_: PDE4 low affinity for rolipram
PEG: Polyethylene glycol
R_L_: Airway resistance

## References

[1] Lee ME, Markowitz J, Lee JO, Lee H (2002). Crystal structure of phosphodiesterase 4D and inhibitor complex (1). FEBS Lett.

[2] Giembycz MA (2000). Phosphodiesterase 4 inhibitors and the treatment of asthma: where are we now and where do we go from here?. Drugs.

[3] Ko WC, Shih CM, Lai YH, Chen JH, Huang HL (2004). Inhibitory effects of flavonoids on phosphodiesterase isozymes from guinea pig and their structure-activity relationships. Biochem Pharmacol.

[4] Hsu HT, Wang WH, Han CY, Chen CN, Chen CM, Ko WC (2013). Inhibitory effects of hesperetin derivatives on guinea pig phosphodiesterases and their ratios between high- and low-affinity rolipram binding. J Pharm Sci.

[5] Zhao Y, Zhang HT, O’Donnell JM (2003). Inhibitor binding to type 4 phosphodiesterase (PDE4) assessed using [^3^H]piclamilast and [^3^H]rolipram. J Pharmacol Exp Ther.

[6] Giembycz MA, Field SK (2010). Roflumilast: first phosphodiesterase 4 inhibitor approved for treatment of COPD. Drug Des Devel Ther.

[7] Abbott-Banner KH, Page CP (2014). Dual PDE3/4 and PDE4 inhibitors: novel treatments for COPD and other inflammatory airway diseases. Basic Clin Pharmacol Toxicol.

[8] Yang YL, Hsu HT, Wang KH, Han CY, Chen CM, Chen CM, Ko WC (2011). Hesperetin-7,3’-*O*-dimethylether selectively inhibits phosphodiesterase 4 and effectively suppresses ovalbumin-induced airway hyperresponsiveness with a high therapeutic ratio. J Biomed Sci.

[9] Hamelmann E, Schwarze J, Takeda K, Oshiba A, Larsen GL, Irvin CG, Gelfand EW (1997). Noninvasive measurement of airway responsiveness in allergic mice using barometric plethysmography. Am J Respir Crit Care Med.

[10] Hantos Z, Brusasco V (2002). Assessment of respiratory mechanics in small animals: the simpler the better?. J Appl Physiol.

[11] Lundblad LK, Irvin CG, Adler A, Bates JH (2002). A reevaluation of the validity of unrestrained plethysmography in mice. J Appl Physiol.

[12] Robichaud A, Savoie C, Stamatiou PB, Lachance N, Jolicoeur P, Rasori R, Chan CC (2002). Assessing the emetic potential of PDE4 inhibitors in rats. Br J Pharmacol.

[13] Robichaud A, Savoie C, Stamatiou PB, Tattersall FD, Chan CC (2001). PDE4 inhibitors induce emesis in ferrets via a noradrenergic pathway. Neuropharmacology.

[14] Ferte J, Kuhnel JM, Chapuis G, Rolland Y, Lewin G, Schwaller MA (1999). Flavonoid-related modulators of multidrug resistance: synthesis, pharmacological activity, and structure-activity relationships. J Med Chem.

[15] Bourne HR, Lichtenstein LM, Melmon KL, Henney CS, Weinstein Y, Shearer GM (1974). Modulation of inflammation and immunity by cyclic AMP. Science.

[16] Robichaud A, Stamatiou PB, Jin SL, Lachance N, MacDonald D, Laliberte F, Liu S, Huang Z, Conti M, Chan CC (2002). Deletion of phosphodiesterase 4D in mice shortens α_2_-adrenoceptor-mediated anesthesia, a behavioral correlate of emesis. J Clin Invest.

[17] Kuehl FA, Zanetti ME, Soderman DD, Miller DK, Ham EA (1987). Cyclic AMP-dependent regulation of lipid mediators in white cells. A unifying concept for explaining the efficacy of theophylline in asthma. Am Rev Respir Dis.

[18] Kammer GM (1988). The adenylate cyclase-cAMP-protein kinase A pathway and regulation of the immune response. Immunol Today.

[19] Moore AR, Willoughby DA (1995). The role of cAMP regulation in controlling inflammation. Clin Exp Immunol.

[20] Kim E, Chun HO, Jung SH, Kim JH, Lee JM, Suh BC, Xiang MX, Rhee CK (2003). Improvement of therapeutic index of phosphodiesterase type IV inhibitors as anti-Asthmatics. Bioorg Med Chem Lett.

[21] Hatzelmann A, Schudt C (2001). Anti-inflammatory and immunomodulatory potential of the novel PDE4 inhibitor roflumilast in vitro. J Pharmacol Exp Ther.

